# Myeloid Pannexin-1 mediates acute leukocyte infiltration and leads to worse outcomes after brain trauma

**DOI:** 10.1186/s12974-020-01917-y

**Published:** 2020-08-20

**Authors:** Joon Ho Seo, Miloni S. Dalal, Frances Calderon, Jorge E. Contreras

**Affiliations:** Department of Pharmacology, Physiology and Neuroscience, New Jersey Medical School, Rutgers University, Medical Sciences Building I-615; 185 S. Orange Avenue, Newark, NJ 07103 USA

**Keywords:** Blood–brain barrier, Hemichannels, Inflammation, Pannexin, Traumatic brain injury

## Abstract

**Background:**

Neuroinflammation is a major component of secondary damage after traumatic brain injury (TBI). We recently reported that pharmacological inhibition of Pannexin-1 (Panx1) channels markedly reduced the inflammatory response after TBI. Panx1 channels have been shown to be important conduits for adenosine 5′-triphosphate (ATP) release and are associated with leukocyte infiltration and pyroptosis. Because Panx1 blockers significantly decrease ATP release and migration of activated microglia and other myeloid cells (such as monocyte-derived macrophages and dendritic cells) in vitro, we hypothesized that myeloid Panx1 channels play a specific role in immune cell infiltration promoting tissue damage following TBI.

**Methods:**

The murine-controlled cortical impact (CCI) model was used on myeloid-specific Panx1 conditional knockout (*Cx3cr1-*Cre*::Panx1*^fl/fl^) mice to determine whether myeloid Panx1 mediates neuroinflammation and brain damage. Immune cell infiltration was measured using flow cytometry. Locomotor and memory functions were measured using the rotarod and Barnes maze test, respectively. The levels of biomarkers for tissue damage and blood–brain barrier leakage were measured using western blot and magnetic resonance imaging. Panx1 channel activity was measured with ex vivo dye uptake assays, using flow cytometry and confocal microscopy.

**Results:**

CCI-injured *Cx3cr1-*Cre*::Panx1*^fl/fl^ mice showed markedly reduced immune cell infiltration to the brain parenchyma compared with *Panx1*^fl/fl^ mice. As expected, Panx1 dependent activity, assessed by dye uptake, was markedly reduced only in myeloid cells from *Cx3cr1-*Cre*::Panx1*^fl/fl^ mice. The expression of biomarkers of tissue damage was significantly reduced in the CCI-injured *Cx3cr1-*Cre*::Panx1*^fl/fl^ mice compared with *Panx1*^fl/fl^ mice. In line with this, magnetic resonance imaging showed reduced blood–brain barrier leakage in CCI-injured *Cx3cr1-*Cre*::Panx1*^fl/fl^ mice. There was also a significant improvement in motor and memory function in *Cx3cr1-*Cre*::Panx1*^fl/fl^ mice when compared with *Panx1*^fl/fl^ mice within a week post-CCI injury.

**Conclusion:**

Our data demonstrate that CCI-related outcomes correlate with Panx1 channel function in myeloid cells, indicating that activation of Panx1 channels in myeloid cells is a major contributor to acute brain inflammation following TBI. Importantly, our data indicate myeloid Panx1 channels could serve as an effective therapeutic target to improve outcome after TBI.

## Introduction

Traumatic brain injury (TBI) affects more than 10 million people worldwide each year. In the United States, close to 50,000 of them do not survive [1]. The pathophysiology of TBI largely consists of two distinct events: primary injury and secondary injury. Primary injury occurs immediately after the impact to the head, and it begets necrotic death of glial cells, neurons, and blood vessels [2–5]. Secondary injury occurs days to weeks later and it accompanies sustained oxidative stress, excitotoxicity, and mitochondrial dysfunction caused by hypoxic-ischemic damage [1]. The injury promotes a neuroinflammatory response that, when sustained (and not resolutive), is shown to negatively contribute to the brain trauma outcome [6]. Activated microglia and infiltrated immune cells can release a plethora of inflammatory mediators that contribute to acute loss of blood–brain barrier integrity and promote further infiltration of peripheral leukocytes to the parenchyma [7]. Limiting the influx of peripheral cells such as neutrophils and monocytes has been shown to be neuroprotective. Depletion of neutrophil or blocking the receptor CD11d improved neurological scores, brain edema, tissue loss, and monocyte activation in animal models of TBI [8, 9]. More recent studies using genetic approaches demonstrated that inhibiting monocyte infiltration by disrupting chemokine signaling, including that of CCL2/CCR2 and CX3CL1/CX3CR1 showed acute protection after TBI in mice models [10, 11].

A critical signaling pathway involved in chemotaxis and infiltration of immune cells is mediated by activation of purinergic receptors (P2X and P2Y) via Adenosine 5′-triphosphate (ATP) [12]. Genetic deletion and pharmacological inhibition of various purinergic receptors have been shown to improve outcomes after experimental models of spinal cord and brain injury [13–16]. This indicates that ATP release and subsequent purinergic signaling may potentially be a therapeutic target for the treatment of traumatic brain injury. Recently, Pannexin 1 (Panx1) channels have been found to be the main conduits of ATP release at the plasma membrane [17]. They have been involved in the induction of inflammation, leukocyte infiltration, and pyroptosis [18–20]. For example, activated THP-1 macrophages release ATP in a Panx1-dependent manner, which activates NLRP3 inflammasome and leads to the secretion of IL-1β [21]. Panx1 channel-dependent release of ATP is required for leukocyte emigration through the endothelium [22], as well as migration of dendritic cells [23] and CD4 T-cells [24]. Previous data from our laboratory has shown that administration of a Panx1 inhibitor, trovafloxacin, improves outcome after TBI and significantly reduced accumulation and infiltration of microglia and macrophages at the injury site [25]. While these data strongly suggest that ATP release via Panx1 channels may play a key role in multiple forms of inflammation, due to ubiquitous expression of Panx1, the specific cell type responsible for the exacerbation of neuroinflammation after TBI remains unknown. In this work, we identified myeloid Panx1 channels as a major player in the leukocyte infiltration triggered by TBI. Genetic deletion of myeloid Panx1 reduces tissue damage, blood–brain barrier leakage and improves behavioral outcomes after brain trauma.

## Materials and methods

### Controlled cortical impact

All procedures that involved animal work were approved by the Institutional Animal Care and Use Committee of Rutgers-New Jersey Medical School. Transgenic mice were group-housed during pre- and postoperative procedures with temperature control (23 °C), 12-h light-dark cycle and ad libitum access to food and water. Sex has a significant effect on outcomes after TBI [26–28]. In the current study, we performed experiments only in male to maximize limited resources and avoid misinterpretation of the results. The 10-week old male mice, weighing at least 25 g, were anesthetized in a mixture of 3~4% isoflurane in oxygen, followed by a maintenance dose of 2~3% in oxygen administered via a nose mask. A rectal temperature probe was used to monitor the body temperature, which was kept at 37 ± 0.5 °C using a heating pad. Local anesthesia (buprenorphine 0.1 mg/kg, subcutaneous injection) was given, followed by a head shave. Animals were then secured on a stereotaxic frame (Stoelting Co. Wood Dale, IL), and a 20-mm midline incision was made over the skull. A unilateral craniectomy was performed between Bregma and Lambda using a hand drill with a 5-mm diameter trephine. Special care was given to stop the drilling when the spongy bone appeared to prevent damage to the dura mater. The bone flap was removed carefully with a pair of Dumont forceps, and using the Impact One (Leica, Concord, Ontario), the animal was given an impact with a 4-mm stainless steel impactor tip, at coordinates AP-2.26 mm, ML + 2.0 mm and depth of 0.65 mm at a rate of 4.0 m/s and a dwell time of 200 ms, at an angle of 0.4°. After the injury, the wound was cleaned and closed with 6-0 nylon sutures, the animal was removed from anesthesia and placed back in a cage over a heating pad, and the righting reflex was monitored for recovery. Sham animals went through the same procedures as CCI-injured animals, except for the craniectomy and the CCI, as it has been shown that craniotomy itself can cause inflammation [29].

### Behavior analyses

#### Rotarod

To assess motor function after injury, a rotarod machine (IITC Life Sciences, Woodland Hills, CA) that has an accelerating rotating cylinder was used. Briefly, mice were trained on the apparatus 3 days (3 trials per day, ITI ~ 30 min) prior to CCI or sham surgery, and the last training day was considered the baseline. The test was performed 1, 3, and 5 days post-injury (DPI), and the latency to fall was measured in each trial.

#### Barnes maze

On 7–11 days post-injury, mice (*n* = 4 for shams and *n* = 8 for CCI) were trained on a spatial reference memory task in the Barnes maze [30–32]. The maze consisted of a white circular platform 100 cm in diameter, elevated 85 cm from the floor with 16 equally spaced holes of 5 cm in diameter along the circumference of the maze. Three visual cues were located on the wall that surrounded the maze. Under one of the potential escape holes, a black plexiglass box was placed for mice to escape. The location of the escape box was consistent for a given mouse but different across the group. The potential intra-maze cues were abolished by rotating the maze in each trial while keeping the relative locations of the escape box to the visual cues constant. To remove olfactory cues, the maze was wiped with 70% ethanol. An incandescent light was placed above the maze to lit up the maze with a light level of ~400 lux.

For habituation, mice were placed in an adaptation box (20 × 15 × 15 cm) in the center of the maze for 1 min, then the box was lifted. The animal was then gently guided to the escape box and when they entered, the escape box covered, and the light was turned off. Mice were kept in the escape box for 2 min and then placed back in the cage. After the habituation period, mice went through the acquisition phase, where they explored the maze until they find the escape box or 3 min has passed, whichever came first. Each mouse performed 2 trials per day (30 min ITI) for 4 consecutive days. On the 5th day, reference memory function was measured by performing a 60-s long probe trial where the escape box was removed and the duration of time mice spent in each of 4 quadrants (target, east, north, south) was measured.

### Western blot analysis

Tissues for western blot analyses were collected on 6 days post-injury. The injured brain tissues from the ipsilateral hemisphere were carefully dissected under a dissecting microscope, and then homogenized in a buffer solution containing M-PER Mammalian protein extraction reagent 5 mM Na_3_VO_4_, 1 mM NaF, 1 mM Na_2_P_2_O_7_, 1 mM Benzamidine, 5 mM EDTA, and HALT Protease Inhibitor Cocktail (Thermo Fisher Scientific, Waltham, MA). Total protein concentration was estimated using a BCA kit (Thermo Fisher Scientific, Waltham, MA). Protein samples were separated by 4–20% SDS-PAGE and transferred to PVDF membrane (BioRad, Hercules, CA). The blots were incubated in Signal Enhancer HIKARI (Nacalai Tesque INC, Japan) containing primary antibodies against alpha II spectrin (Santa Cruz Biotechnology, Dallas, TX), MMP9 (NeuroMab, San Diego, CA), IgG (Thermo Fisher Scientific, Waltham, MA) or GAPDH (Cell Signaling, Danvers, MA). Molecular mass was estimated with a pre-stained protein marker with a standard molecular weight range (BioRad, Hercules, CA). Blots were developed using an ECL kit (Thermo Fisher Scientific, Waltham, MA) and visualized by LAS-3000 Imaging System (Fuji, Cambridge, MA). Densitometric analyses were performed using ImageJ.

### Flow cytometry analysis of infiltrating leukocyte and microglia

To isolate microglia and infiltrating monocytes, brains were freshly dissected at 3 DPI and cells were dissociated to be analyzed by flow cytometry. Briefly, mice were anesthetized with ketamine and then transcardially perfused with an ice-cold saline solution containing 20 U/mL heparin. Ipsilateral hemisphere from CCI-injured brains or sham-injured brains were carefully dissected, minced (~1 mm in diameter) in cold Hanks’ Balanced Salt Solution using scalpels, and then the tissue was passed through 70 μm cell strainer. Brain tissue was centrifuged at 300–400 g at 4 °C for 5 min, followed by digestion in 2 U/mL Liberase TL (Sigma Aldrich, city, state) for 1 hour at 37 °C. Subsequently, cells were washed with 666 U/mL DNAse (Worthington Biochemical Corp., Lakewood, NJ), passed through 100 μm cell strainer, followed by centrifugation at 300–400*g* at 4 °C for 5 min. Cells were then identified by surface expression of CD11b, CD45, Ly6C, and Ly6G. To block the FCγRs, cells were preincubated with anti-mouse CD16/CD32 antibody (BD Biosciences, San Jose, CA) for 15 min at 4 °C, and then incubated with PerCP-Cy5.5-anti CD11b antibody (BD Biosciences, San Jose, CA), PE-Cy7-antiCD45 antibody (Thermo Fisher Scientific, Waltham, MA), BV510-anti Ly6C antibody (Biolegend, San Diego, CA) and APC-anti Ly6G antibody (Thermo Fisher Scientific, Waltham, MA) for 20 min at 4 °C in the dark. Cells were analyzed by LSRII flow cytometer (BD Biosciences, San Jose, CA), and then analyzed by FACS Diva software. Gating strategy is described in Supplementary Figs. 2 and 3.

### Assessment of Panx1 channel activity via dye uptake

To isolate microglia and infiltrating monocytes, brains from *Cx3cr1*^EGFP/Cre^ :: *Panx1*^fl/fl^ mice were freshly dissected at 3 DPI and cells were dissociated to be analyzed by flow cytometry. Briefly, mice were anesthetized with ketamine and then transcardially perfused with an ice-cold saline solution containing 20 U/mL heparin. Undamaged brain hemispheres from sham animals or ipsilateral hemispheres from CCI-injured brains were carefully dissected, minced (~1 mm in diameter) in cold Hanks’ Balanced Salt Solution using scalpels, and then the tissue was passed through 70 μm cell strainer. Brain tissue was centrifuged at 300–400*g* at 4 °C for 5 min, followed by digestion in 2 U/mL Liberase TL (Sigma Aldrich, city, state) for 1 h at 37 °C. Subsequently, cells were washed with 666 U/mL DNAse (Worthington Biochemical Corp., Lakewood, NJ), passed through 100 μm cell strainer, followed by centrifugation at 300–400*g* at 4 °C for 5 min. Cells were resuspended in 300 μl DMEM/1%BSA, and then co-incubated with BzATP (300 μM, Sigma) and 1 μM To-Pro-3 for 30 min, followed by flow cytometry analysis. Cells were analyzed by LSRII flow cytometer (BD Biosciences, San Jose, CA), and then analyzed by FACS Diva software. Gating strategy is described in Supplementary Figs. 2 and 3. For the analysis by confocal microscopy, cells were co-incubated with BzATP (300 μM, Sigma), 1 μM To-Pro-3, and DAPI (1:10,000). Time-series acquisitions were obtained for a 25-min time period, every 5 mins. Cells that were positive for DAPI were excluded from the analysis. To-Pro-3 dye fluorescence emission (642/661) was detected at 37 °C using appropriate lasers.

### Magnetic resonance imaging (MRI) analysis of mouse brain after CCI

MRI scanning was performed at 6 DPI using M2™ Compact High-Performance MRI (1 T) scanner (Aspect, Israel). A custom-made MRI compatible head holder was used to secure the animal, which was anesthetized in a mixture of 3~4% isoflurane in oxygen, followed by a maintenance dose of 2~3% in oxygen administered via a nose mask. Physiological monitoring (respiration and temperature) was performed throughout the procedures to ensure mice’s wellbeing and acquisition reproducibility. The body temperature was kept at 37 ± 0.5 °C using a custom-made heating pad. Mice were initially scanned with a Fast Spin Echo to determine the brain, ventricle, and wound size. To measure blood–brain barrier leakage, mice were pre-scanned with Gradient Spin Echo, dosed with Magnevist (0.1 mmol/kg, tail vein), and then scanned again, followed by volumetric analysis to determine the volume of dye leaked into the parenchyma.

### Statistical analysis

Values are represented as mean ± standard deviation. Comparisons between groups were made using linear regression analysis using the treatment and the interaction between treatment and time as factors (Rotarod and Barnes maze). Paired Student’s *t* test (Magnevist uptake), one-way ANOVA (western blot, flow cytometry) plus post hoc test were used as appropriate. The values were expressed as the means ± SD. The differences with *P* < 0.05 were considered statistically significant.

## Results

### Myeloid Panx1 channels promote leukocyte infiltration after traumatic brain injury

Our previous study revealed that inhibition of Panx1 channels reduced the migration of microglia in vitro. Furthermore, our in vivo data showed that the infiltration of CD68 positive cells (activated microglia/macrophages) to the injury site was reduced following TBI when mice were treated with the Panx1 inhibitor, trovafloxacin [25]. To determine if Panx1 activity in a specific immune cell population underlies infiltration and migration after brain TBI, we created a transgenic mice in which Panx1 is selectively deleted in cells expressing the chemokine receptor CX3CR1 (*Cx3cr1-*Cre*::Panx1*^fl/fl^); this includes microglia, monocytes, and macrophages. *Panx1*^fl/fl^ mice, which mice carry the transgene cassette, but do not have CX3CR1 deletion, were used as controls [33]. The absence of *Panx1* was confirmed using FACS followed by detection of mRNA by qRT-PCR (Supplementary Figure 1).

**Fig. 1:**
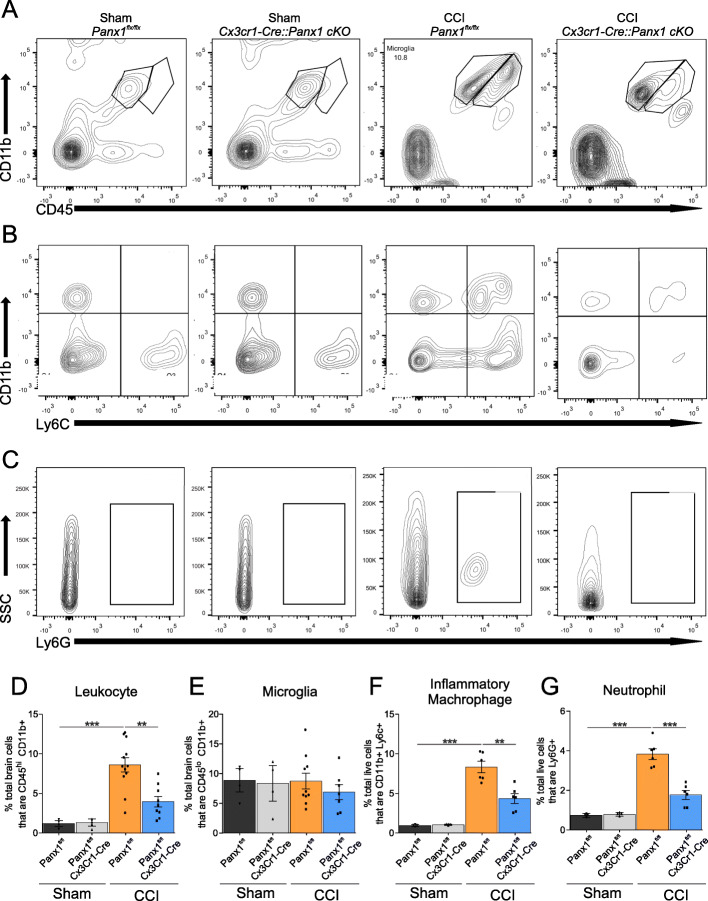
Infiltration of peripheral leukocytes, including monocytes and neutrophils, are reduced in *Cx3cr1*-Cre::*Panx1*^fl/fl^ mice compared to *Panx1*^fl/fl^ mice. Representative flow cytometry plots for **(a)** peripheral leukocytes and microglia, **(b)** inflammatory monocytes, and **(c)** neutrophils 3-days post-injury. Quantification indicates that the number of **(d)** CD45^hi^ CD11b^+^ peripheral leukocytes (**P < 0.01, ***P < 0.001, one-way ANOVA (F_3,23_ = 13.31), Tukey’s post hoc test), **(f)** CD45^hi^ and Ly6c inflammatory monocytes (**P < 0.01, ***P < 0.001, one-way ANOVA (F_3,12_ = 19.62), Tukey’s post hoc test) and **(g)** neutrophils (**P < 0.01, ***P < 0.001, one-way ANOVA (F_3,12_ = 27.31), Tukey’s post hoc test) were reduced in *Cx3cr1-*Cre*::Panx1*^fl/fl^ mice compared to *Panx1*^fl/fl^ mice after TBI. **(e)** shows that the number of microglia are not changed between the genotypes. Values are expressed as mean (±SD) mean fluorescence intensity relative to the background. *n* = 4 to 12 mice per group

To characterize the immune cell population that infiltrates the brain from resident microglia after TBI, we analyzed ipsilateral brain tissue using flow cytometry in sham and CCI-injured mice 3 days post-CCI using two cell surface markers: CD11b and CD45 [34]. Figure 1a shows a clear population of CD11b^+^ and CD45^lo^ cells from sham *Panx1*^fl/fl^ and *Cx3cr1-*Cre*::Panx1*^fl/fl^ mice, which correspond to microglia [35, 36]. CCI-injury, however, produced a robust infiltration of CD11b^+^ CD45^hi^ in *Panx1*^fl/fl^ mice, which is consistent with peripheral leukocytes infiltrating the parenchyma as previously reported [35, 36]. Strikingly, there was a significant reduction of CD11b^+^ CD45^hi^ leukocytes in CCI-injured *Cx3cr1-*Cre*::Panx1*^fl/fl^ mice (Fig. 1d, Sham *Panx1*^fl/fl^: 1.28 ± 0.53; Sham *Cx3cr1-*Cre*::Panx1*^fl/fl^; 1.24 ± 0.64; CCI *Panx1*^fl/fl^; 8.61 ± 3.11; CCI *Cx3cr1-*Cre::*Panx1*^fl/fl^: 3.95 ± 2.03; *n* = 4 to 12 per group; **P < 0.001, ***P < 0.0001), indicating that Panx1 channel deletion in myeloid cells markedly reduced leukocyte infiltration. To narrow down the identity of the infiltrating leukocytes, we additionally tested for Ly6c and Ly6g expression to identify pro-inflammatory monocytes and neutrophils, respectively. Figure 1b and c shows that CCI induced a robust infiltration of both CD11b^+^ CD45^hi^ Ly6c^+^ cells (monocytes) and CD11b^+^ CD45^hi^ Ly6g^+^ cells (neutrophils). In contrast, *Cx3cr1-*Cre*::Panx1*^fl/fl^ mice showed significantly less number of both monocytes (Fig. 1f, Sham *Panx1*^fl/fl^: 0.89 ± 0.14; Sham *Cx3cr1-*Cre*::Panx1*^fl/fl^; 0.99 ± 0.11; CCI *Panx1*^fl/fl^; 8.33 ± 1.78; CCI *Cx3cr1-*Cre::*Panx1*^fl/fl^: 4.35 ± 1.51; *n* = 4 to 6 per group; **P < 0.001, ***P < 0.0001) and neutrophils (Fig. 1g, Sham *Panx1*^fl/fl^: 0.68 ± 0.13; Sham *Cx3cr1-*Cre*::Panx1*^fl/fl^; 0.79 ± 0.13; CCI *Panx1*^fl/fl^; 3.83 ± 0.66; CCI *Cx3cr1-*Cre::*Panx1*^fl/fl^: 1.77 ± 0.54; *n* = 4 to 6 per group; ***P < 0.001) when compared with *Panx1*^fl/fl^ mice. Collectively, these data suggest that myeloid Panx1 channels promote the infiltration of peripheral immune cells, including pro-inflammatory monocytes, and neutrophils early after CCI-injury.

**Fig. 2:**
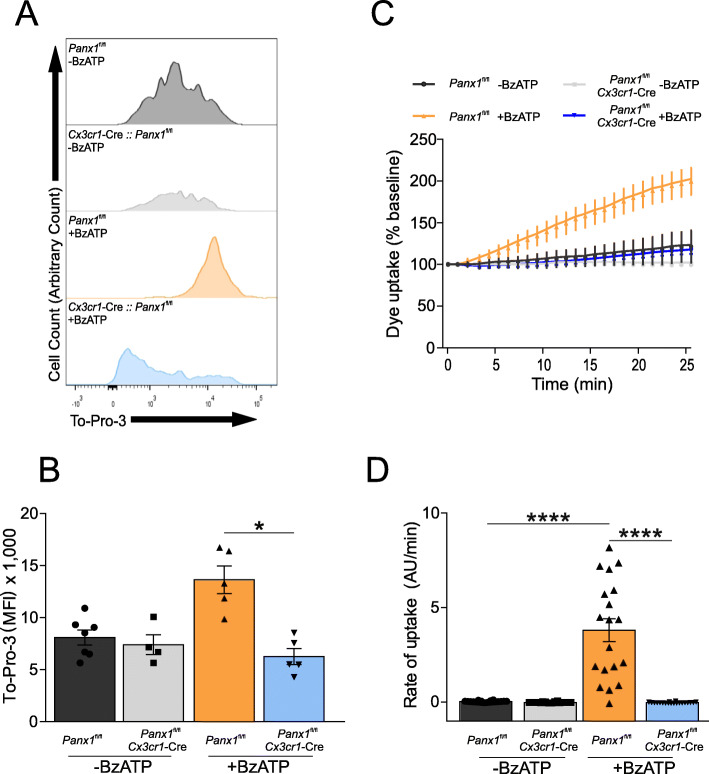
Panx1 activity is reduced in myeloid cells (EGFP positive) from *Cx3cr1*-Cre::*Panx1*^fl/fl^ mice compared to the *Panx1*^fl/fl^ mice. BzATP-induced To-Pro 3 uptake via Panx1 activation was assessed using flow cytometry (**a** and **b**) and confocal microscopy (**c** and **d**). At 3-days post-injury, the ipsilateral cortex was isolated and dissociated to obtain Cx3Cr1^+^ cells. **a** Representative example of flow cytometry analysis for each group showing To-Pro 3 uptake. **b** Quantification of mean fluorescence intensity of the different groups (*n* = 4 or 6 mice per group; One-way ANOVA (F_3,17_ = 11.56), Tukey’s post hoc test). **c** Kinetic of To-pro-3 uptake in isolated Cx3Cr1^+^ cells measured by confocal microscopy. **d** Rate of To-pro-3 uptake. At least 20 cells from three separate experiments were used to obtain the rate. One-way ANOVA (F_3,96_ = 48.87) and Tukey’s post hoc test. *P > 0.05, **** *P* < 0.0001

To correlate the role of myeloid Panx1 in leukocyte infiltration with channel function, we then assessed dye uptake (TO-PRO-3) in isolated myeloid cells from CCI-injured mice at 3 DPI upon stimulation with BzATP. Previous reports have shown that BzATP promotes activation of purinergic receptors, which via intracellular signaling activate Panx1 channels [37]. To facilitate microglia and macrophages isolation and dye uptake quantification, we generated a *Panx1*^fl/fl^ and *Cx3cr1-*Cre*::Panx1*^fl/fl^ mice expressing EGFP in myeloid cells*.* As shown in Fig. 2a and b, flow cytometry analysis revealed that CCI-injury produces BzATP-induced TO-PRO-3 uptake in EGFP positive myeloid cells from CCI-injured *Panx1*^fl/fl^ mice but not from *Cx3cr1-*Cre*::Panx1*^fl/fl^ mice (Fig. 2a and b, -BzATP *Panx1*^fl/fl^: 8.81 ± 1.87; -BzATP *Cx3cr1-*Cre*::Panx1*^fl/fl^; 7.38 ± 1.90; +BzATP *Panx1*^fl/fl^; 13.62 ± 2.95; +BzATP *Cx3cr1-*Cre::*Panx1*^fl/fl^: 6.26 ± 1.72; *n* = 4 to 5 per group; *P < 0.05). Consistently, time-lapse measurements of TO-PRO-3 uptake rates in EGFP positive myeloid cells from CCI-injured *Panx1*^fl/fl^ mice were significantly higher than those obtained from Cx3cr1*-*Cre*::Panx1*^fl/fl^ mice (Fig. 2c and d, -BzATP *Panx1*^fl/fl^: 0.02 ± 0.01; -BzATP *Cx3cr1-*Cre*::Panx1*^fl/fl^; 0.01 ± 0.026; +BzATP *Panx1*^fl/fl^; 3.81 ± 2.61; +BzATP *Cx3cr1-*Cre::*Panx1*^fl/fl^: 0.02 ± 0.01; *n* = 25 cells per group from *n* = 3 mice ; ****P < 0.0001). Importantly, these results linked the neuroinflammatory role of myeloid Panx1 with channel function.

### Deletion of myeloid Panx1 channels ameliorates behavioral deficits and tissue damage caused by traumatic brain injury

**Fig. 3:**
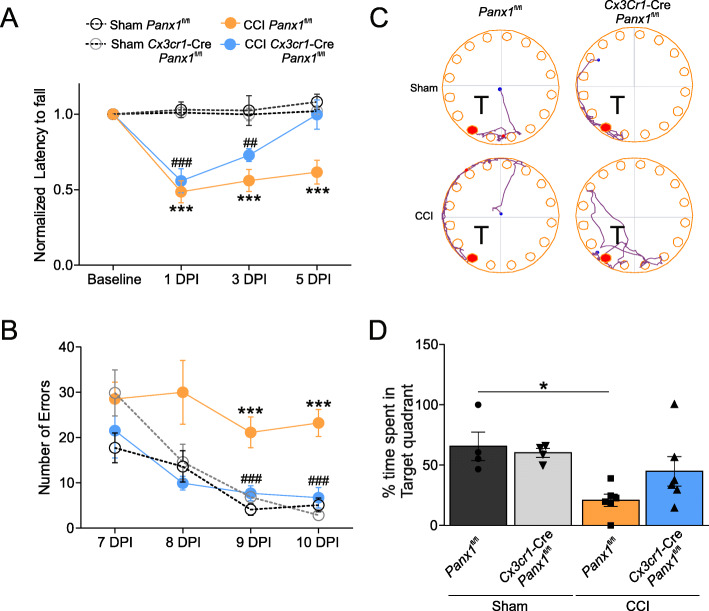
Deletion of myeloid Panx1 improves behavioral outcomes after TBI. **a** The rotarod test was used to evaluate latency to fall in sham *Panx1*^fl/fl^ (grey open circles) and *Cx3cr1*-Cre::*Panx1*^fl/fl^ mice (black open circles), as well as CCI *Panx1*^fl/fl^ (orange closed circles) and *Cx3cr1*-Cre::*Panx1*^fl/fl^ mice (blue closed circles). The test was performed 1-, 3-, and 5-days post-injury. Values are represented as mean ± SD (*n* = 8 per group). Linear regression analysis was used to determine the statistical difference between different groups at each time point (*P* values calculated for treatment and treatment*time). ***P < 0.0001, sham *Panx1*^fl/fl^ vs CCI *Panx1*^fl/fl^; ###*P* < 0.0001, ##*P* < 0.001, sham *Cx3cr1*-Cre::*Panx1*^fl/fl^ vs. CCI *Cx3cr1*-Cre::*Panx1*^fl/fl^. **b** Mice were tested in Barnes maze 7 days after sham or CCI. The average number of errors per day during the 4-day acquisition period was measured in sham *Panx1*^fl/fl^ (grey open circles) and *Cx3cr1*-Cre::*Panx1*^fl/fl^ mice (black open circle), as well as CCI *Panx1*^fl/fl^ (orange closed circle) and *Cx3cr1*-Cre::*Panx1*^fl/fl^ mice (blue closed circles). Values are represented as mean ± SD (*n* = 4 to 8 per group). Linear regression analysis was used to determine the statistical difference between different groups at each time point (*P* values calculated for treatment and treatment*time). ***P < 0.0001, sham *Panx1*^fl/fl^ vs CCI *Cx3cr1*-Cre::*Panx1*^fl/fl^; ###*P* < 0.0001, sham *Panx1*^fl/fl^ day 1 vs sham *Panx1*^fl/fl^ day 3 and day 4. **c** Representative image of mouse track during the probe trial (“T” represents target quadrant). **d** Bar charts of the proportion of time spent in the target quadrant during the probe trial (*P < 0.05; 2-way ANOVA followed by Tukey’s multiple comparisons test)

We previously showed that pharmacological inhibition of Panx1 channels improved motor function after CCI-injury [25]. To test whether Panx1 channels in myeloid cells were responsible for locomotor impairment after TBI, CCI-injured Panx1^fl/fl^ and *Cx3cr1-*Cre*::Panx1*^fl/fl^ mice were subjected to the rotarod test at 1, 3, and 5 days post-injury (DPI). CCI-injury produced a significant reduction in motor coordination at 1 DPI, indicated by ~50% reduction in the latency to fall compared with sham-operated animals in *Panx1*^fl/fl^ and *Cx3cr1-*Cre*::Panx1*^fl/fl^ mice (Fig. 3a). CCI-injured *Panx1*^fl/fl^ mice performance remained impaired during the entire duration of the study (5 DPI). In contrast, CCI-injured *Cx3cr1-*Cre*::Panx1*^fl/fl^ mice showed progressive recovery with longer latency to fall at 3 and 5 DPI with no statistical difference between CCI-injured *Cx3cr1-*Cre*::Panx1*^fl/fl^ mice and sham-operated animals at 5 DPI (Fig. 3a, *n* = 8 per group; ***P < 0.0001, sham *Panx1*^fl/fl^ vs CCI *Panx1*^fl/fl^; ###*P* < 0.0001, ##*P* < 0.001, sham *Cx3cr1-*Cre*::Panx1*^fl/fl^ vs. CCI *Cx3cr1-*Cre*::Panx1*^fl/fl^). Control sham-injured mice, in both *Cx3cr1-*Cre*::Panx1*^fl/fl^ and *Panx1*^fl/fl^ groups, as expected, showed no change in latency to fall from the baseline. These data suggest that abolished Panx1 activity in myeloid cells prevents TBI-induced motor deficits.

To assess the role of Panx1 channels on spatial memory function after TBI, sham and CCI-injured mice were subjected to Barnes maze task with visual cues, where they learned the location of the escape hole for 4 consecutive days, followed by a probe trial on 11 DPI. As shown in Fig. 3b, *Panx1*^fl/fl^ and *Cx3cr1-*Cre*::Panx1*^fl/fl^ sham mice showed a reduction in the number of attempts finding the escape hole (number of errors) during the acquisition phase, indicating that the mice are learning the task. In contrast, when the mice were subjected to CCI-injury, only *Cx3cr1-*Cre*::Panx1*^fl/fl^ mice, but not *Panx1*^fl/fl^, mice showed a reduction in the number of errors during the 4-day testing period (Fig. 3b, *n* = 4 for sham group, *n* = 6 for CCI group; ***P < 0.0001, sham *Panx1*^fl/fl^ vs CCI *Cx3cr1*-Cre::*Panx1*^fl/fl^; ###*P* < 0.0001, sham *Panx1*^fl/fl^ day 1 vs sham *Panx1*^fl/fl^ day 3 and day 4). This indicates that the deletion of myeloid Panx1 improves the retention of spatial memory following brain injury (Fig. 3b). Subsequently, the retention of spatial reference memory was assessed with a probe trial at 11 DPI, where the escape box was removed and mice were allowed to search for its prior location [38]. Unlike CCI-injured *Panx1*^fl/fl^ mice, which showed no preference of the target quadrant, CCI-injured *Cx3cr1-*Cre*::Panx1*^fl/fl^ showed comparable preference to the target quadrant (denoted as “T” in Fig. 3c) as the sham groups (Fig. 3c and d, Sham *Panx1*^fl/fl^: 65.67 ± 23.60; Sham *Cx3cr1-*Cre*::Panx1*^fl/fl^; 20.92 ± 12.57; CCI *Panx1*^fl/fl^; 60.25 ± 7.29; CCI *Cx3cr1-*Cre::*Panx1*^fl/fl^: 44.92 ± 30.20; *n* = 4 for sham group, *n* = 6 for CCI group, *P < 0.05). These results suggest that *Cx3cr1-*Cre*::Panx1*^fl/fl^ mice retain spatial reference memory after CCI injury, unlike *Panx1*^fl/fl^ mice.

**Fig. 4:**
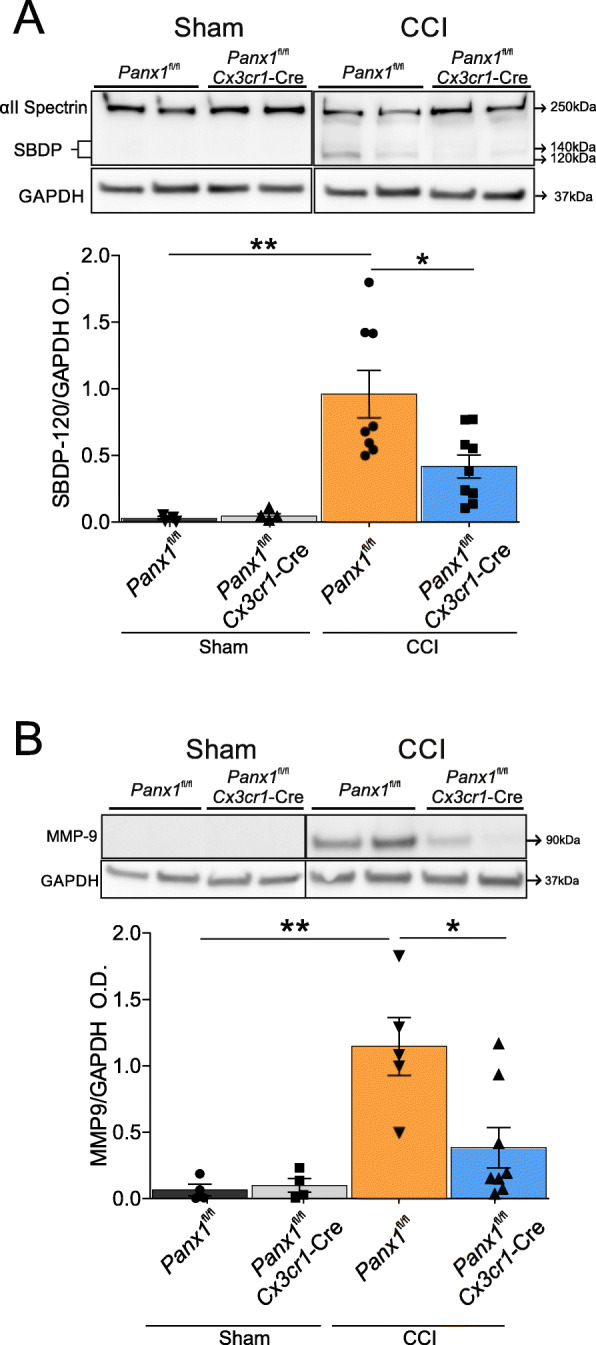
Panx1 deletion in myeloid cells reduces biomarkers of TBI. Brain tissue was isolated from ipsilateral cortex from each group and protein expression levels were determined by western blot at 6-days post-injury: **a** Representative western blots images showing α II spectrin and its spectrin breakdown product (SBDP 120 kDa and SBDP 140 kDa) levels at 6 days post-injury in both sham and CCI-injured *Cx3cr1*-Cre::*Panx1*^fl/fl^ and *Panx1*^fl/fl^ mice. The bottom western blot corresponds to the GAPDH, which was used as a housekeeping gene. The graph shows densitometric quantification of SBDP 120 kDa normalized to GAPDH values. Values are represented as mean ± SD (*n* = 4 to 8 per group). One-way ANOVA (F_3,19_ = 8.237) and Tukey’s post hoc test. **b** Representative western blots images showing MMP-9 levels at 6 days post-injury in both sham and CCI-injured *Cx3cr1*-Cre::*Panx1*^fl/fl^ and *Panx1*^fl/fl^ mice. The bottom western blot corresponds to the GAPDH, which was used as a housekeeping gene. The graph shows densitometric quantification of MMP-9 normalized to GAPDH values. Values are represented as mean ± SD (*n* = 4 to 8 per group). Values are expressed as the mean ± SD optical density relative to GAPDH. *P < 0.05, **P < 0.01, One-way ANOVA (F_3,17_ = 8.864) and Tukey’s post hoc test.

To determine whether the observed rescue of behavioral deficits in *Cx3cr1-*Cre*::Panx1*^fl/fl^ mice correlated with reduced tissue damage, we analyzed protein levels of previously established biomarkers of TBI, namely α-II spectrin and MMP9 [25, 39, 40]. α-II spectrin is a structural protein abundant in neurons that is cleaved into signature fragments of 120 and 140 kDa (SBDP-120 and SBDP-140) by proteases involved in necrotic and apoptotic cell death [39]. As shown in Fig. 4a, sham surgery did not cause the breakdown of full-length spectrin, whereas CCI-injury produced a significant increase in spectrin breakdown product (SBDP-120) in *Panx1*^fl/fl^ mice. In line with our hypothesis, the levels of SBDP-120 were significantly reduced in CCI-injured *Cx3cr1-*Cre*::Panx1*^fl/fl^ mice. This suggests that myeloid Panx1 channels indirectly enhanced neuronal death after brain injury (Fig. 4a, Sham *Panx1*^fl/fl^: 0.041 ± 0.044; Sham *Cx3cr1-*Cre*::Panx1*^fl/fl^; 0.064 ± 0.035; CCI *Panx1*^fl/fl^; 0.96 ± 0.50; CCI *Cx3cr1-*Cre::*Panx1*^fl/fl^: 0.41 ± 0.26; *n* = 4 to 8 per group; **P < 0.01, *P < 0.05). Additionally, when we analyzed protein levels of MMP9, a matrix metalloproteinase associated with neuroinflammation and blood-brain barrier disruption [41], we observed that CCI produced a significant increase in MMP9 in *Panx1*^fl/fl^ mice, but not in *Cx3cr1-*Cre*::Panx1*^fl/fl^ animals (Fig. 4b, Sham *Panx1*^fl/fl^: 0.056 ± 0.078; Sham *Cx3cr1-*Cre*::Panx1*^fl/fl^; 0.086 ± 0.096; CCI *Panx1*^fl/fl^; 1.147 ± 0.483; CCI *Cx3cr1-*Cre::*Panx1*^fl/fl^: 0.16 ± 0.15; *n* = 4 to 5 per group, *P < 0.05). No MMP9 was detected in brain samples from sham mice *Panx1*^fl/fl^ and *Cx3cr1-*Cre*::Panx1*^fl/fl^. Taken together, these results indicate that the genetic deletion of myeloid Panx1 improves behavioral recovery and limits acute tissue damage after CCI injury.

### Deletion of myeloid Panx1 channels attenuates blood–brain barrier disruption after CCI

**Fig. 5:**
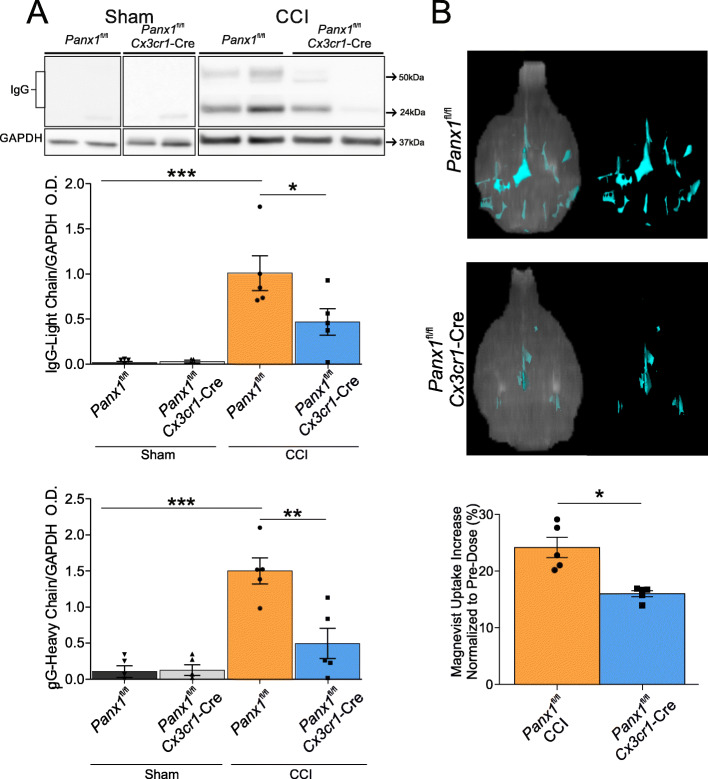
Panx1 deletion in myeloid cells reduces blood–brain barrier leakage after TBI. **a** Immunoglobulin (IgG) heavy chain and light chain, markers for blood–brain barrier damage, are reduced in *Cx3cr1*-Cre::*Panx1*^fl/fl^ mice (*n* = 5) compared with *Panx1*^fl/fl^ mice (*n* = 5). Light chain: *P < 0.05, ***P < 0.001, One-way ANOVA (F_3, 14_ = 12.04) and Tukey’s post hoc test. Heavy chain: **P < 0.01, ***P < 0.001, One-way ANOVA (F_3,14_ = 16.89) and Tukey’s post hoc test. Values are expressed as mean ± SD optical density relative to GAPDH. Each dot represents an individual animal. **b** Magnevist present into the parenchyma was calculated by subtracting basal Magnevist in the parenchyma from post-dose. Unpaired Student’s two-tailed *t* test (*t* = 4.361, df = 8, *P* = 0.0024). Values are expressed as the mean difference in (±SD) Magnevist signal before and after dose. Each dot represents an individual animal. *P < 0.05.

To test whether Panx1 channels in myeloid cells contribute to blood–brain barrier disruption after CCI injury, we performed western blot analysis against IgG in brain samples from *Panx1*^fl/fl^ and *Cx3cr1-*Cre*::Panx1*^fl/fl^ mice at 6 DPI. Figure 5 shows that the levels of heavy and light chain IgG are not noticeable in the sham cortex and that CCI induces significant elevation of both proteins in the *Panx1*^fl/fl^ mice. Conversely, CCI-injured *Cx3cr1-*Cre*::Panx1*^fl/fl^ mice show a significant decrease in protein levels of heavy and light IgG chains compared with *Panx1*^fl/fl^ mice. In the healthy brain, IgG is not found in sham *Panx1*^fl/fl^ and *Cx3cr1-*Cre*::Panx1*^fl/fl^ mice. In contrast, CCI-injury produced robust detection of IgG in the ipsilateral hemisphere of *Panx1*^fl/fl^ mice when compared with sham mice (Fig. 5a, Light chains: Sham *Panx1*^fl/fl^: 0.017 ± 0.028; Sham *Cx3cr1-*Cre*::Panx1*^fl/fl^; 0.030 ± 0.030; CCI *Panx1*^fl/fl^; 1.00 ± 0.43; CCI *Cx3cr1-*Cre::*Panx1*^fl/fl^: 0.47 ± 0.33, *n* = 4 to 5 per group; *P < 0.05, ***P < 0.001; heavy chains: Sham *Panx1*^fl/fl^: 0.16 ± 0.16; Sham *Cx3cr1-*Cre*::Panx1*^fl/fl^; 0.13 ± 0.15; CCI *Panx1*^fl/fl^; 1.50 ± 0.40; CCI *Cx3cr1-*Cre::*Panx1*^fl/fl^: 0.50 ± 0.50, *n* = 4 to 5 per group; **P < 0.01, ***P < 0.001).

To further investigate blood–brain barrier dysfunction post-CCI, we used dynamic contrast-enhanced magnetic resonance imaging (DCE-MRI) to quantify the volume of Magnevist, a contrast agent that leaked into the brain parenchyma after CCI injury [42, 43]. At 6 DPI, CCI-injured mice were scanned using a 1 T High-Performance MRI. Figure 5b shows that at this time point, there is a significant reduction of contrast volume in *Cx3cr1-*Cre*::Panx1*^fl/fl^ mice when compared with *Panx1*^fl/fl^ mice (Fig. 5b, *Panx1*^fl/fl^; 24.18 ± 4.00; *Cx3cr1-*Cre::*Panx1*^fl/fl^: 16.02 ± 1.23, *n* = 5 per group, *P < 0.05), indicating that myeloid Panx1 channels promote persistent blood–brain barrier disruption following CCI. These results are consistent with the western blot data showing that myeloid deletion of Panx1 reduced the IgG leakage.

## Discussion

We previously showed that pharmacological inhibition of Panx1 channels improved outcomes and reduced biomarkers for neuroinflammation in CCI-injured mice [25]. Here, we build upon these findings and identify Panx1 channels in myeloid cells (microglia and monocytes) as major players in mediating acute neuroinflammation after TBI. Our results indicate that genetic deletion of myeloid Panx1 reduced infiltration of peripheral leukocytes including inflammatory monocytes and neutrophils, decreased post-TBI memory and locomotor dysfunction, and ameliorated tissue damage and blood–brain barrier dysfunction after brain trauma. Thus, our data demonstrates a critical role for the activity of Panx1 channels in myeloid cells following TBI.

Recent studies indicate that myeloid Panx1 is critical for the progression of joint and nerve-injury pain, likely by attenuating microglial and macrophage activation at the spinal cord [44, 45]. A major finding from our work is that myeloid Panx1 promotes acute infiltration of pro-inflammatory cells after brain trauma, which consequently impacts the neuroinflammatory response and outcomes after TBI. Previous work showed that the infiltration of peripheral immune cells into the parenchyma is a major hallmark of traumatic brain injury [1]. Specifically, monocytes and neutrophils are among the first cells to infiltrate the brain, where they substantially contribute to neuroinflammation and tissue damage, primarily by secreting pro-inflammatory molecules, damage-associated molecular patterns (DAMPs), and various matrix metalloproteinases, leading to blood–brain barrier breakdown [46].

One of the molecules that are critical for migration and infiltration of immune cells is ATP, which is released by multiple cell types upon injury to activate purinergic signaling [47]. Recently, compelling evidence indicated that ATP release via Panx1 channels is critical for the emigration of circulating leukocytes. For instance, the deletion of endothelial Panx1 channels decreased local ATP release, leukocyte adhesion, and extravasation through blood vessels when stimulated with TNF-α [22]. Dendritic cells lacking Panx1 display impaired ATP release and homing to lymph nodes [23], suggesting a role of these channels in their cellular migration. In line with these observations, our data showed that the deletion of Panx1 channels in myeloid cells decreased the infiltration of leukocytes at the injury site after TBI. Thus, we speculate that the deletion of Panx1 in monocytes/macrophages impairs the migration of these cells. This might attenuate the overall neuroinflammatory response, consequently reducing the infiltration of other immune cells like neutrophils. This notion is consistent with several lines of evidence that showed the role of infiltrating monocytes in the recruitment of various leukocytes, including neutrophils and T-cells, following brain trauma [48, 49]. For example, signaling by chemokine (C-C motif) ligand 2 (CCL2) to the chemokine (C-C motif) receptor 2 (CCR2) is a key regulator of brain infiltration by monocytes after brain trauma. Genetic deletion of the CCR2 receptor in monocytes reduced the overall leukocyte infiltration to the injury site post-CCI [11]. It also decreased tissue loss and axonal damage at the early stages after CCI-injury. Studies targeting the CX3CR1 fractalkine receptor, which is expressed in both microglia and infiltrating monocytes show similar results to those described for CCR2 receptors upon brain injury [50]. Because Panx1 channel signaling is associated with leukocyte migration, it is not surprising that our results resemble those described for the above chemokine receptors.

A growing body of evidence suggests that Panx1 is involved in different pathologies that affect the nervous system [44, 51–53]. In a rodent model of brain ischemia, pharmacological blockade and genetic ablation of Panx1 channels have been shown to improve behavioral outcomes and reduce infarct volumes [52, 53]. Pharmacological inhibition of Panx1 alleviated tissue damage in rats with intracerebral hemorrhage [54, 55]. Interestingly, the role of Panx1 channels in neuroinflammation has not yet been linked with brain damage. Neuroinflammation and brain damage directly correlate with behavioral outcomes in TBI [11, 49, 56, 57]. Our data showed that CCI-injured mice lacking Panx1 in myeloid cells displayed reduced protein levels of spectrin breakdown product (SBDP) and matrix metalloproteinase-9 (MMP-9), which are well-known TBI biomarkers for tissue damage and neuroinflammation [38]. This might also explain the less severe blood–brain barrier dysfunction observed in CCI-injured mice lacking myeloid Panx1 and suggests that myeloid Panx1 channels drive blood–brain barrier dysfunction after brain injury. We also reported a considerable improvement in motor and neurocognitive functions in myeloid Panx1-deleted animals. This is consistent with the previous report that linked neuroinflammation with acute spatial memory deficits in various animal models of brain injury and neurodegenerative diseases [30, 32, 58, 59]. These results confirm and extend our previous finding that pharmacological inhibition of Panx1 with trovafloxacin reduces neuroinflammation, blood–brain barrier leakage and improves motor function after TBI.

While it is evident that panx1 channel blockers and deletion of myeloid Panx1 reduce inflammation and promote neuroprotection in short-term studies, the role of Panx1 channels in chronic post-TBI phases has yet to be investigated. The present study, however, provides substantial evidence to suggest that myeloid Panx1 is a key player in the acute neuroinflammatory response triggered by TBI. Our results coupled with our previous pharmacological findings support a model whereby blockade of Panx1 channels improves outcome after TBI [25] and further strengthens the potential for cell-specific Panx1 targeted strategies to treat brain injuries.

## Conclusions

Here, we show that the deletion of myeloid Panx1 reduced infiltration of pro-inflammatory immune cells, reduced brain damage and improved memory and locomotor outcomes in a mouse model of TBI. Previously, we reported that systemic pharmacological inhibition of Panx1 channels markedly reduced neuroinflammation and improved outcomes after TBI. In the current study, we have taken a genetic approach to narrow down the deleterious actions of Panx1 activation to only a handful of myeloid cells. Our results indicate that the activity of Panx1 channels in myeloid cells contributes to the degree of neuroinflammation and the severity of brain damage following TBI. We propose that selective inhibition of Panx1 channels in these cells can be used to develop new therapeutic approaches to treat inflammation after TBI.

## Supplementary information

**Additional file 1: Figure S1.** Generation of the conditional myeloid Panx1 knockout mice. Mice harboring Panx1 deletion were generated using the cre-loxP system [33]. Panx1tm1a(KOMP)Wtsi mice containing 2 loxP sequences flanking exon 3 of the Panx1 gene were obtained from the KOMP repository, and then bred to mice harboring FLP1 recombinase gene under the control of the human ACTB promotor (Jax mice: ACTB:FLPe B6N, stock number 019100) to generate homozygous *Panx1*^fl/fl^ mice. These mice were crossed with C57BL/6 J mice that express Cre recombinase under the direction of the Cx3cr1 promoter (Jax mice: B6N (Cg)-Cx3cr1tm1.1(cre)Jung/J, stock number 025524) and then backcrossed 8 generations to yield the myeloid conditional knockout *Cx3cr1*-Cre::*Panx1*^fl/fl^ mice. To generate myeloid reporter mice with Panx1 deletion mutation, we crossed *Panx1*^fl/fl^ and *Cx3cr1*-Cre::*Panx1*^fl/fl^ mice with Cx3Cr1^EGFP/WT^ mice. **(A)** Targeting strategy for conditional deletion of Panx1. Peripheral macrophages were isolated using FACS from **(B)** Floxed and **(C)** conditional knockout mice. **(D)** mRNA expression of wild-type *panx1* in peritoneal macrophages. Statistical significance was evaluated using a One-way ANOVA (F_3,3_ = 68.38); *n* = 3 per group. * *P* < 0.05

**Additional file 2: Figure S2.** Gating strategy for brain immune cells Flow cytometry analysis showing brain-resident microglia, peripheral leukocytes, inflammatory monocytes and neutrophils.

**Additional file 3: Figure S3.** Gating strategy for To-Pro-3 uptake Flow cytometry analysis showing Cx3Cr1-EGFP cells and To-Pro-3 uptake.

## Data Availability

The datasets analyzed during the current study are available from the corresponding author on reasonable request
