# Supplementary material for: Myeloid Pannexin-1 mediates acute leukocyte infiltration and leads to worse outcomes after brain trauma
Source: J Neuroinflammation. 2020 Aug 20;17:245. doi: 10.1186/s12974-020-01917-y (PMC7441665; doi:10.1186/s12974-020-01917-y)
Supplement: Supplementary file 1 — Additional file 1: Figure S1. Generation of the conditional myeloid Panx1 knockout mice. Mice harboring Panx1 deletion were generated using the cre-loxP system [33]. Panx1tm1a(KOMP)Wtsi mice containing 2 loxP sequences flanking exon 3 of the Panx1 gene were obtained from the KOMP repository, and then bred to mice harboring FLP1 recombinase gene under the control of the human ACTB promotor (Jax mice: ACTB:FLPe B6N, stock number 019100) to generate homozygous Panx1fl/fl mice. These mice were crossed with C57BL/6 J mice that express Cre recombinase under the direction of the Cx3cr1 promoter (Jax mice: B6N (Cg)-Cx3cr1tm1.1(cre)Jung/J, stock number 025524) and then backcrossed 8 generations to yield the myeloid conditional knockout Cx3cr1-Cre::Panx1fl/fl mice. To generate myeloid reporter mice with Panx1 deletion mutation, we crossed Panx1fl/fl and Cx3cr1-Cre::Panx1fl/fl mice with Cx3Cr1EGFP/WT mice. (A) Targeting strategy for conditional deletion of Panx1. Peripheral macrophages were isolated using FACS from (B) Floxed and (C) conditional knockout mice. (D) mRNA expression of wild-type panx1 in peritoneal macrophages. Statistical significance was evaluated using a One-way ANOVA (F3,3 = 68.38); n = 3 per group. * P < 0.05 [file 12974_2020_1917_MOESM1_ESM.pdf]

Supplementary Figure 1

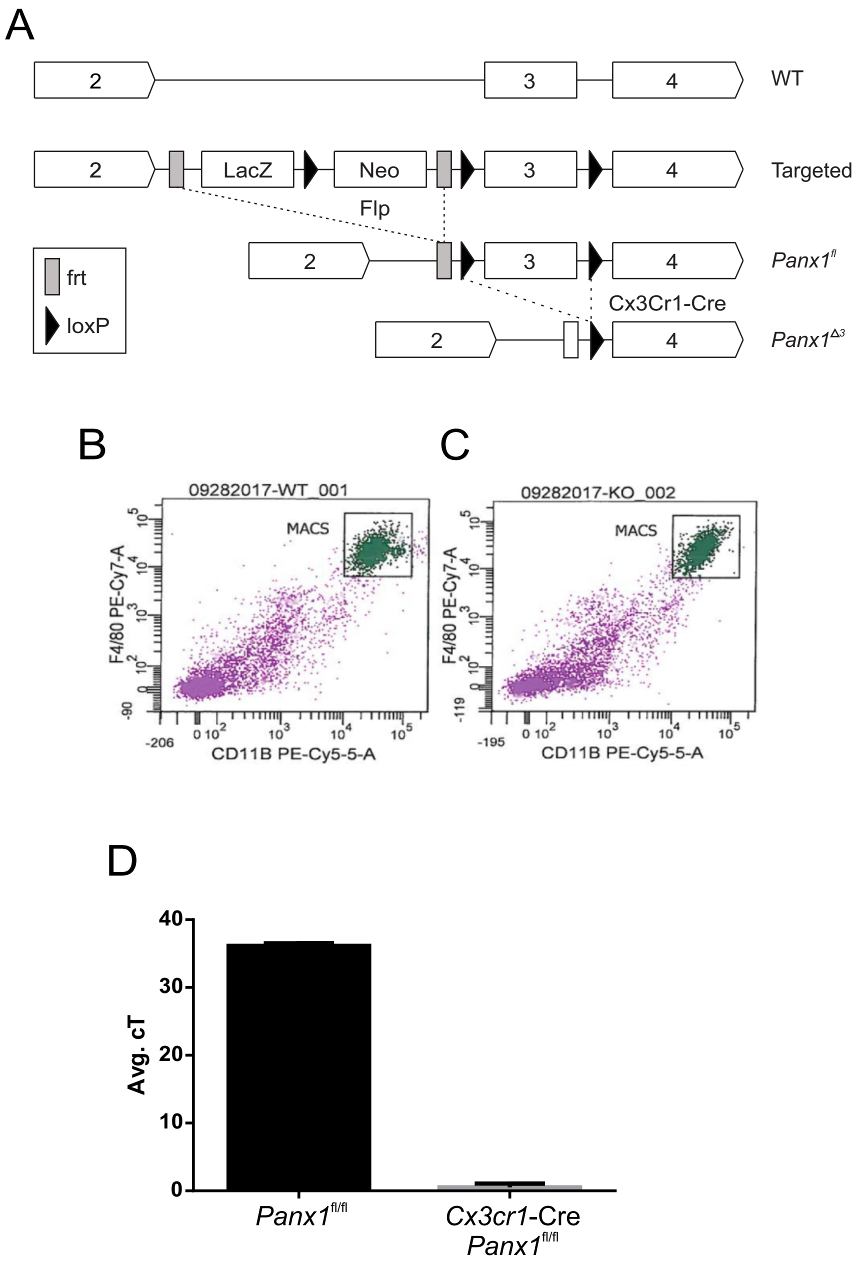

Supplementary Fig. 1 Generation of *Cx3cr1*-Cre::*Panx1*$^{fl/fl}$ transgenic mice

Mice harboring Panx1 deletion was generated using the cre-loxP system (34). Panx1tm1a(KOMP)Wtsi mice containing 2 loxP sequences flanking exon 3 of the Panx1 gene were obtained from KOMP repository, and then bred to mice harboring FLP1 recombinase gene under the control of the human ACTB promotor (Jax mice: ACTB:FLPe B6N, stock number 019100) to generate homozygous Panx1$^{fl/fl}$ mice. These mice were crossed with C57BL/6J mice that express Cre recombinase under the direction of the Cx3cr1 promoter (Jax mice: B6N(Cg)-Cx3cr1tm1.1(cre)Jung/J, stock number 025524) and then backcrossed 8 generations to yield the myeloid conditional knockout *Cx3cr1*-Cre::*Panx1*$^{fl/fl}$ mice. To generate myeloid reporter mice with Panx1 deletion mutation, we crossed Panx1fl/fl and *Cx3cr1*-Cre::*Panx1*$^{fl/fl}$ mice with *Cx3Cr1*$^{EGFP/EGFP}$ mice. **(A)** Targeting strategy for conditional deletion of Panx1. Peripheral macrophages were isolated using FACS from **(B)** Floxed and **(C)** conditional- knockout mice. **(D)** mRNA expression of wild-type panx1 in peritoneal macrophages. Statistical significance was evaluated using one way ; n ≥ 3 per group. N.D. Non-detected
