# Supplementary material for: Myeloid Pannexin-1 mediates acute leukocyte infiltration and leads to worse outcomes after brain trauma
Source: J Neuroinflammation. 2020 Aug 20;17:245. doi: 10.1186/s12974-020-01917-y (PMC7441665; doi:10.1186/s12974-020-01917-y)
Supplement: Supplementary file 2 — Additional file 2: Figure S2. Gating strategy for brain immune cells Flow cytometry analysis showing brain-resident microglia, peripheral leukocytes, inflammatory monocytes and neutrophils. [file 12974_2020_1917_MOESM2_ESM.pdf]

Supplementary Figure 2

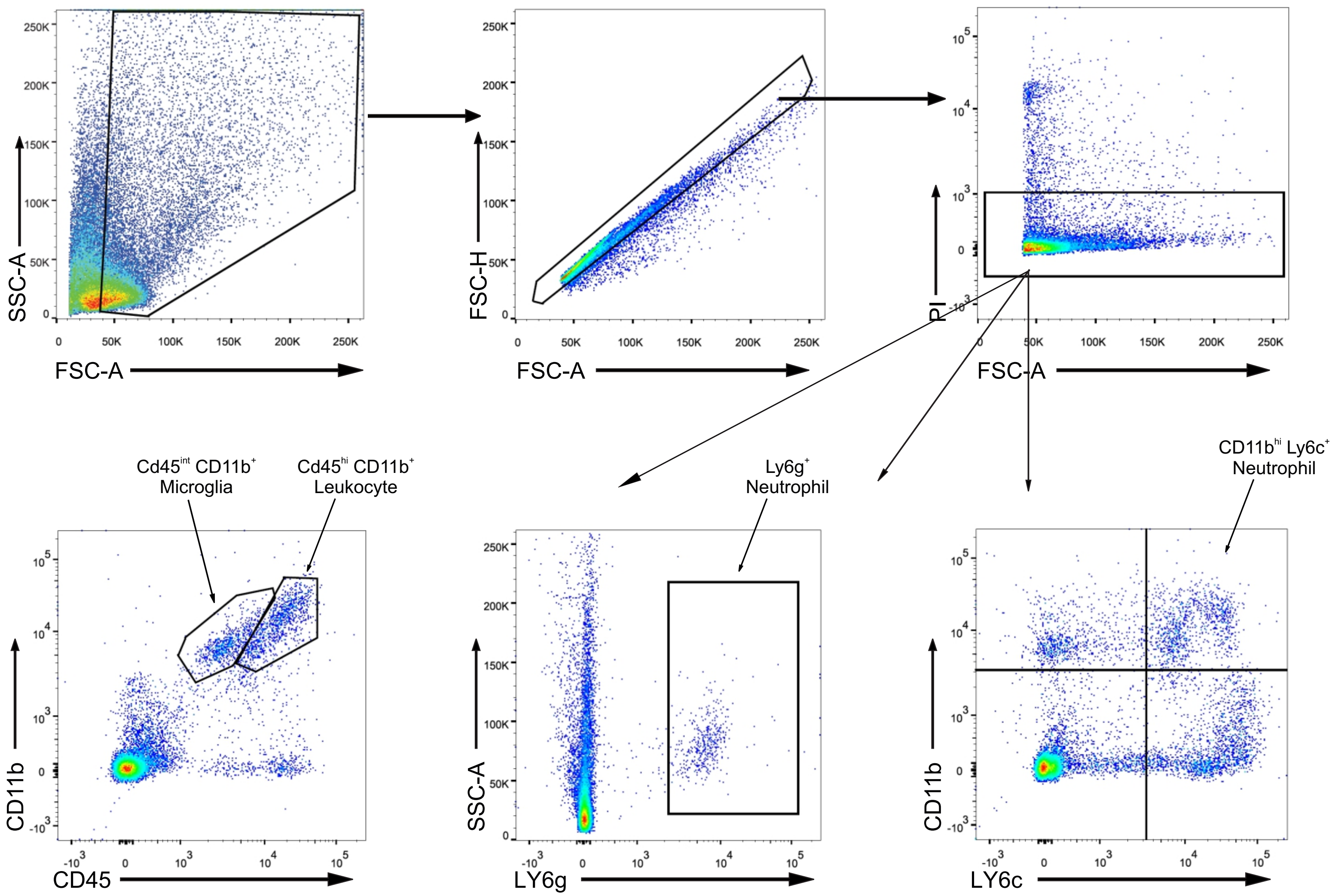

Supplementary Fig. 2 Gating strategy for brain immune cells
Flow cytometry analysis showing brain-resident microglia, peripheral leukocytes, inflammatory monocytes and neutrophils.
