# Supplementary material for: Myeloid Pannexin-1 mediates acute leukocyte infiltration and leads to worse outcomes after brain trauma
Source: J Neuroinflammation. 2020 Aug 20;17:245. doi: 10.1186/s12974-020-01917-y (PMC7441665; doi:10.1186/s12974-020-01917-y)
Supplement: Supplementary file 3 — Additional file 3: Figure S3. Gating strategy for To-Pro-3 uptake Flow cytometry analysis showing Cx3Cr1-EGFP cells and To-Pro-3 uptake. [file 12974_2020_1917_MOESM3_ESM.pdf]

Supplementary Figure 3

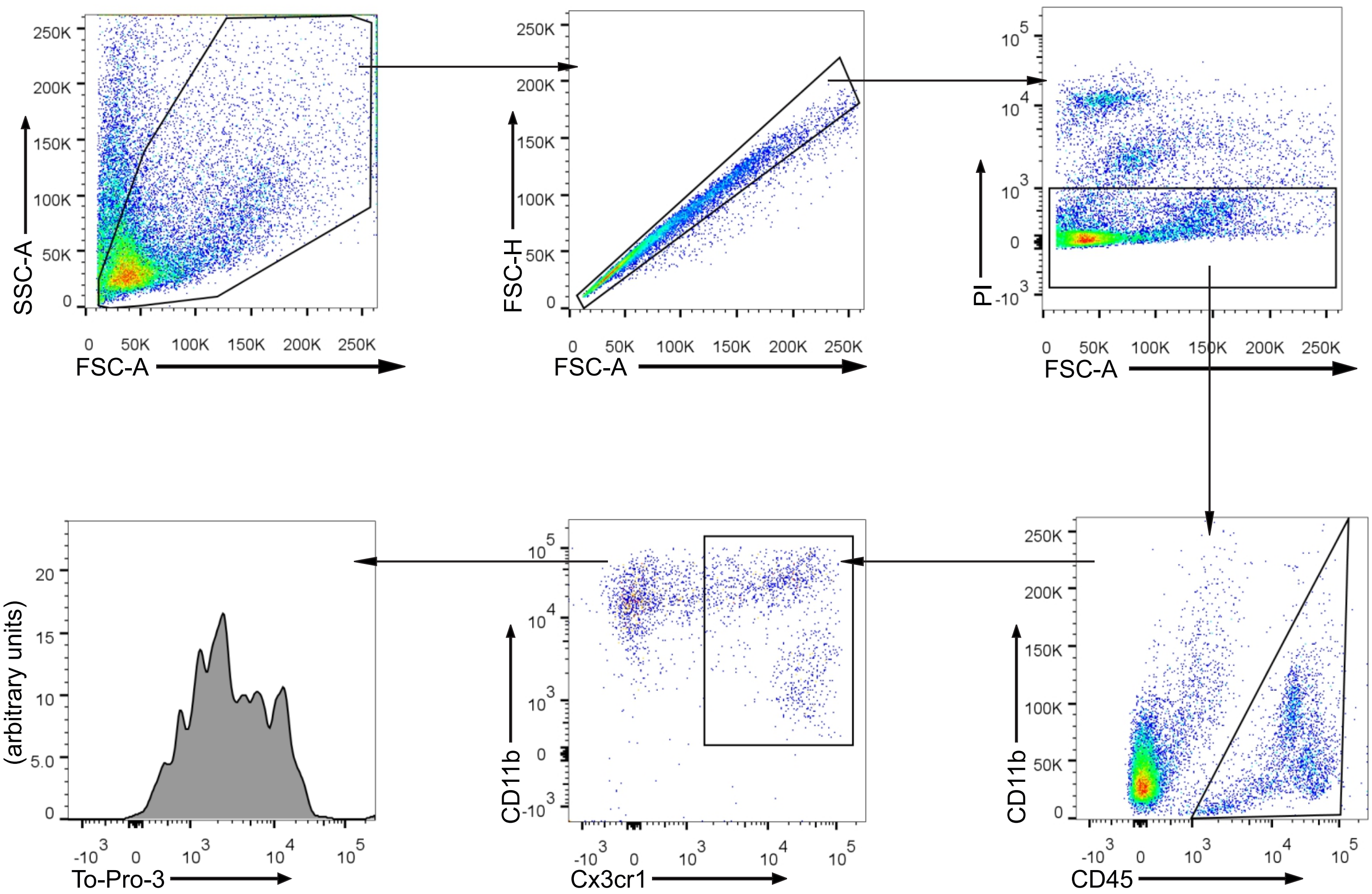

Supplementary Fig. 3 Gating strategy for To-Pro-3 uptake
Flow cytometry analysis showing Cx3Cr1-EGFP cells and To-Pro-3 uptake.
